# Supplementary material for: Investigating COVID-19 Vaccine Impact on the Risk of Hospitalisation through the Analysis of National Surveillance Data Collected in Belgium
Source: Viruses. 2022 Jun 16;14(6):1315. doi: 10.3390/v14061315 (PMC9228783; doi:10.3390/v14061315)
Supplement: Supplementary file 1 [file viruses-14-01315-s001.zip › viruses-1732665-supplementary.pdf]

Table S1: definition of the different vaccination status in the Surge Capacity (SC) survey and in the vaccine coverage data sets.

Vaccination status	Correspondence in the SC survey data set	Correspondence in the vaccine coverage data set
Unvaccinated	No vaccine dose administered	Not included in the data set
Partially vaccinated	1 of 2 doses (BNT162b2, mRNA-1273, ChAdOx1)	Category A
Fully vaccinated (primary course)	1 of 1 dose (Ad26.COV2), or 2 of 2 doses (BNT162b2, mRNA-1273, ChAdOx1)	Categories B and C
Boosted*	1 of 1 dose (Ad26.COV2) + 1 booster, or 2 of 2 doses (BNT162b2, mRNA-1273, ChAdOx1) + 1 booster	Category E
Unknown	The hospital was not aware of the vaccination status at time of reporting	Not applicable

(*) While booster doses started to be administered on September 22, 2021, this category was added in the SC survey on December 8, 2021.

Table S2: approximation of vaccine impact on the risk of hospitalisation through the estimation of risk ratios (RR) based on the analyses of hospitalisation surveillance data collected in Belgium. We here report RR values estimated for different age categories, as well as for different periods and/or vaccination schemes. Of note, in Belgium, hospitalised patients having had their booster jab were only registered as "boosted" at their admission from December 8, 2021. Therefore, we are only able to make the distinction between "only primary course completed" and "boosted" vaccination schemes for the second half of the study period (15/12/22-28/02/22). In addition to RR estimates, we also report associated 95% confidence intervals (20). When using a sliding window, confidence intervals were computed by estimating the 2.5 and 97.5% quantiles.

	25-34	35-44	45-54	55-64	65-74	75-84	85+	overall[1]	overall[2]
**06/10/2021 - 14/12/2021 - at least primary course**	0.16 [0.13-0.19]	0.09 [0.08-0.11]	0.11 [0.10-0.12]	0.14 [0.13-0.16]	0.22 [0.20-0.24]	0.38 [0.35-0.42]	0.42 [0.37-0.48]	0.31 [0.29-0.32]	0.61 [0.58-0.63]
**15/12/2021 - 28/02/2022 - at least primary course**	0.38 [0.32-0.44]	0.25 [0.22-0.29]	0.19 [0.17-0.22]	0.16 [0.14-0.18]	0.19 [0.17-0.21]	0.30 [0.27-0.33]	0.54 [0.48-0.60]	0.36 [0.35-0.38]	0.50 [0.49-0.52]
**15/12/2021 - 28/02/2022 - only primary course**	0.33 [0.28-0.39]	0.27 [0.23-0.31]	0.27 [0.23-0.32]	0.37 [0.32-0.42]	0.74 [0.66-0.83]	1.07 [0.95-1.20]	0.91 [0.80-1.05]	0.37 [0.35-0.38]	0.43 [0.41-0.45]
**15/12/2021 - 28/02/2022 - boosted *vs* unvaccinated**	0.47 [0.39-0.57]	0.24 [0.20-0.28]	0.14 [0.12-0.17]	0.11 [0.10-0.13]	0.14 [0.13-0.15]	0.23 [0.21-0.26]	0.48 [0.43-0.54]	0.36 [0.34-0.38]	0.56 [0.54-0.58]
**15/12/2021 - 28/02/2022 - boosted *vs* primary course**	0.97 [0.82-1.15]	0.56 [0.47-0.66]	0.33 [0.29-0.38]	0.20 [0.18-0.22]	0.16 [0.15-0.18]	0.22 [0.21-0.24]	0.51 [0.47-0.55]	0.69 [0.66-0.71]	0.85 [0.82-0.87]
**06/10/2021 - 28/02/2022 - at least primary course**	0.26 [0.23-0.29]	0.15 [0.13-0.16]	0.14 [0.13-0.15]	0.15 [0.14-0.16]	0.20 [0.19-0.21]	0.33 [0.31-0.36]	0.49 [0.45-0.53]	0.32 [0.31-0.33]	0.54 [0.53-0.56]
**Sliding window* - at least primary course**	0.19 [0.12-0.64]	0.10 [0.08-0.41]	0.13 [0.06-0.30]	0.15 [0.10-0.23]	0.21 [0.11-0.25]	0.35 [0.17-0.49]	0.42 [0.22-0.66]	0.31 [0.19-0.53]	0.54 [0.35-0.70]
**Sliding window** - boosted *vs* unvaccinated**	0.44 [0.29-0.60]	0.27 [0.16-0.36]	0.20 [0.10-0.24]	0.13 [0.09-0.17]	0.13 [0.08-0.21]	0.23 [0.09-0.35]	0.50 [0.18-0.62]	0.40 [0.21-0.50]	0.53 [0.43-0.61]

Overall RR estimates were obtained by considering several age categories together: overall RR estimates[1] were obtained when considering all age categories together, and overall RR estimates[2] were obtained when only considering the age categories reported in the present table (i.e. only people from 25 years old). (*) RR estimates associated with the "at least primary course completed" vaccination scheme and averaged from daily estimates obtained when using a retrospective 4-weeks sliding window on the entire study period (06/10/21-28/02/22). (**) RR estimates associated with the "boosted" vaccination scheme and averaged from daily estimates obtained when using a retrospective 4-weeks sliding window on the second half of the study period (15/12/21- 28/02/22).
